# Enhancing emotional health and engagement in Chinese English language learners: an approach from teachers’ autonomy- supportive behavior, teachers’ harmony, and peer support in a two-sample study

**DOI:** 10.3389/fpsyg.2024.1356213

**Published:** 2024-03-18

**Authors:** Liu Yang

**Affiliations:** International School, Jinan University, Guangzhou, Guangdong, China

**Keywords:** autonomy-supportive behavior, Chinese education, emotional health, English language learners, peer support, student engagement, Teachers’ Harmony, teaching methods

## Abstract

**Background:**

In the evolving landscape of Chinese education, understanding the factors that influence the emotional health and engagement of English language learners is increasingly vital. Against this backdrop, our study delves into how teachers’ autonomy-supportive behavior, teachers’ harmony, and peer support impact these key educational outcomes.

**Aim:**

This study investigates the roles of teachers’ autonomy-supportive behavior, teachers’ harmony, and peer support in influencing the emotional health and engagement of English language learners in China.

**Method:**

Involving a diverse sample of 68 English Language Teachers and their 389 students from various Chinese universities, the study leverages a convenience sampling method.

**Results:**

Key findings indicate that students’ emotional health is predominantly influenced by peer support, while student engagement is significantly affected by a combination of teachers’ autonomy-supportive behavior, peer support, and teachers’ harmony. These outcomes highlight the importance of both teacher behavior and peer relationships in educational settings, underscoring their crucial roles in enhancing student well-being and engagement. The study’s methodology, incorporating a diverse sample from multiple educational institutions and a comprehensive analytical approach, offers robust insights. However, the limitations of convenience sampling and reliance on self-reported data necessitate a careful interpretation of the findings.

**Implications:**

Implications from this research are vital for educational policy and practice, emphasizing the need for interventions that enhance teacher-student relationships and foster supportive peer environments. This study adds to the body of knowledge on factors influencing emotional health and engagement among English language learners, advocating for a comprehensive approach in educational strategies and interventions.

## 1 Introduction

In an increasingly interconnected world, the educational experiences and challenges of English Language Learners (ELLs) demand a nuanced understanding. This article aims to unravel the complex tapestry of factors influencing the emotional health and academic engagement of ELLs. It interweaves a discussion on the roles of teachers’ autonomy-supportive behavior, the nurturing concept of Teachers’ Harmony, the dynamic impact of peer support, and the unique educational journey of Chinese ELLs within the Confucian value system (Yim et al., 2013; Tan, 2017; Lim and Thien, 2020).

Central to this exploration is the concept of Teachers’ Autonomy-Supportive Behavior, rooted in Self-Determination Theory (Ryan and Deci, 2000; Yang et al., 2022). This approach highlights the pivotal role of educators in creating a learning environment that fosters autonomy, competence, and relatedness. Such an environment not only cultivates intrinsic motivation and engagement but also bolsters students’ emotional well-being, setting a foundation for deeper, more meaningful learning experiences.

Parallel to the influence of teachers is the equally significant realm of peer interactions. Students’ Peer Support emerges as a critical component of the educational ecosystem, offering both emotional sustenance and collaborative learning opportunities (Vargas-Madriz and Konishi, 2021). These peer dynamics, viewed through the lens of Social Identity Theory, are instrumental in shaping students’ emotional health and engagement, particularly as they navigate the intricacies of language and cultural adaptation (John et al., 2018).

Complementing these student-centered factors is the concept of Teachers’ Harmony, a delicate balance that intertwines empathy with authority, and flexibility with structure. This harmony is not only essential for the well-being of educators but also reverberates through their interactions with students, influencing academic outcomes. In environments where Confucian values are predominant, such as in China, Teachers’ Harmony aligns seamlessly with cultural expectations of respect and collective harmony, enriching the learning experience (Li, 2013).

The journey of Chinese ELLs brings unique challenges and insights, especially within the high-pressure, Confucian-influenced educational milieu (Wang, 2023). This context underscores the importance of culturally responsive teaching and the need for educational practices that support emotional stability and academic engagement (Han, 2022). Understanding the specific hurdles faced by these learners illuminates the broader narrative of ELL education, highlighting the need for tailored approaches that consider cultural nuances and individual student needs.

This article, therefore, will begin with the literature revision to support the proposed hypotheses, followed by a description of the method applied, through a quantitative approach based in self-reported questionnaires, and a statistical analysis of the data, that allow to obtain empirical findings, which could be further discussed in light of the previous literature.

## 2 Theoretical development

### 2.1 Emotional health and academic engagement in Chinese ELLs

The constructs of emotional health and academic engagement are pivotal in understanding the educational experiences of English Language Learners (ELLs), particularly in China. Emotional health in educational settings refers to the psychological well-being of students, encompassing aspects like self-esteem, stress management, coping strategies, and overall emotional stability. Academic engagement, on the other hand, relates to the degree of students’ involvement, interest, and commitment to their academic work. It includes behavioral engagement (participation in academic and extracurricular activities), emotional engagement (positive and negative reactions to teachers, classmates, and school), and cognitive engagement (investment in learning and self-regulation).

In the context of Chinese ELLs, these constructs gain added significance due to several challenges faced by these learners. Firstly, language barriers can lead to feelings of frustration, anxiety, and social isolation, impacting emotional health. Secondly, cultural differences may result in difficulties in understanding and integrating into the social and educational norms of the learning environment. Thirdly, the pressure of academic performance, often heightened by societal and familial expectations in China, can exacerbate stress and affect engagement.

Studies have highlighted the importance of addressing the emotional health of ELLs to improve their academic engagement. For instance, research indicates that emotional well-being is a predictor of academic success among ELLs, as students who are emotionally healthy are more likely to be actively engaged in their learning (Roeser et al., 2000; Wang and Eccles, 2012). Additionally, interventions aimed at improving the emotional health of ELLs, such as counseling and peer support programs, have been found to have a positive impact on their academic engagement (Pekrun et al., 2017).

Furthermore, understanding the unique cultural context of Chinese ELLs is essential. The Confucian heritage culture, which emphasizes respect for authority, collectivism, and the importance of education, shapes the educational experiences and expectations of these learners (Li, 2002). This cultural background influences how they perceive and respond to educational challenges, including language learning.

In summary, the emotional health and academic engagement of Chinese ELLs are deeply intertwined and influenced by language barriers, cultural differences, and academic pressures, as well as by teachers’ pedagogical approaches, peer’s behavior and teachers’ individual characteristics. A global model that integrates these relevant components will be proposed and tested in the present research.

### 2.2 Teachers’ autonomy-supportive behavior

Teachers’ Autonomy-Supportive Behavior is a pedagogical approach rooted in the principles of Self-Determination Theory (Deci and Ryan, 2013). Self-Determination Theory emphasizes the importance of autonomy, competence, and relatedness in fostering intrinsic motivation. In the educational context, Teachers’ Autonomy-Supportive Behavior involves providing students with the freedom to explore, make choices, and engage in self-directed learning, thereby nurturing their innate psychological needs.

According to Reeve (2016), Teachers’ Autonomy-Supportive Behavior refers to the extent to which teachers allow students latitude during learning activities. This includes detecting and nurturing students’ needs, interests, and preferences, and offering opportunities in the classroom for students to use their motivations to direct their learning activities (Yang et al., 2022). This approach is directly associated with greater use of learning strategies by students and is mediated by students’ perceptions of teacher support (Brandisauskiene et al., 2023).

The influence of Teachers’ Autonomy-Supportive Behavior extends to various aspects of student development. For instance, it has been linked to learners’ resilience and engagement, particularly in higher education settings. Reeve (2016) and Aelterman et al. (2019) further elaborate on the concept, describing autonomy-supportive behaviors as crucial in shaping students’ motivation to learn. They classify teaching styles into two motivating categories (autonomy-supportive and structuring) and two demotivating categories (controlling and chaotic) (Moè and Katz, 2020).

Students’ perceptions of teacher autonomy support are multifaceted, involving aspects of learning support, emotional support, and ability support (Hu et al., 2023). Furthermore, teacher motivation, including autonomy-supportive behaviors, is known to correlate with student motivation, highlighting the reciprocal nature of teacher-student interactions in educational settings (Slemp et al., 2020).

In the realm of physical education, research has shown that Teachers’ Autonomy-Supportive Behaviors not only promote autonomous motivation but also enhance knowledge structures and motor skills learning (Behzadnia et al., 2019). These behaviors are also critical in enhancing positive student behavior and reducing misbehavior, mediated by advantageous comparison and non-responsibility (Hsu et al., 2021).

The positive relationship between Teachers’ Autonomy-Supportive Behavior and student engagement is moderated by students’ perceived equity (Brandisauskiene et al., 2023). Moreover, in comprehensive school reform contexts, autonomy-supportive teacher behaviors have been identified as key elements in fostering a conducive learning environment (Bozack et al., 2008).

In summary, Teachers’ Autonomy-Supportive Behavior, underpinned by Self-Determination Theory, plays a pivotal role in enhancing student engagement, well-being, and intrinsic motivation (Black and Deci, 2000; Ma, 2021). This approach, by acknowledging and supporting the psychological needs of autonomy, competence, and relatedness, significantly contributes to the emotional health and engagement of students, especially English Language Learners, in diverse educational settings.

### 2.3 Teachers’ harmony

As classes and educational institutions constitute the proximal context for learning, teachers’ personal well-being seems a relevant factor for satisfying experiences. Teachers’ Harmony, particularly in relation to psychological harmony (Ren et al., 2009), focuses on creating a balanced and harmonious educational environment that benefits both teachers and students (Delle Fave et al., 2016). Psychological harmony can be defined as a state of inner peace and balance, crucial for emotional well-being and effective functioning (Garcia et al., 2014). In the context of education, Teachers’ Harmony involves cultivating a classroom atmosphere where there is a balance between structure and flexibility, authority and empathy, and challenge and support (Delle Fave et al., 2022).

The relevance of Teachers’ Harmony in protecting teachers’ psychological well-being and improving student outcomes is significant (Sun et al., 2016). A harmonious environment helps teachers to manage stress and maintain a positive mental state, which in turn affects their teaching effectiveness and their ability to form strong, supportive relationships with students (Di Fabio and Tsuda, 2018). A harmonious teacher-student relationship can lead to better learning outcomes, as students feel more supported, valued, and motivated (Moe and Katz, 2022).

Furthermore, the impact of Teachers’ Harmony on student engagement and emotions is particularly noteworthy under the Confucian values approach, which emphasizes harmony as a fundamental principle (Yim et al., 2013). Confucianism values the cultivation of moral virtues, respect for authority, and the importance of education in personal and societal development (Zhang and Zhou, 2008). In this context, Teachers’ Harmony aligns with the Confucian emphasis on harmonious relationships and respectful interactions, contributing to a positive and conducive learning environment (Wang et al., 2020). This approach fosters students’ emotional well-being, engagement, and academic success.

### 2.4 Students’ peer support

Students’ Peer Support in educational contexts is a significant factor in fostering emotional health and engagement among learners. This concept involves the assistance, encouragement, and emotional support that students provide to each other, creating a network of mutual aid and understanding within the educational environment. Peer support plays a crucial role in enhancing the overall well-being of students, as it is associated with improvements in mental health, including increased happiness, self-esteem, effective coping skills, and reductions in depression, loneliness, and anxiety. These benefits have been observed across diverse groups, including university students, non-student young adults, and ethnic/sexual minorities (Richard et al., 2022).

However, the impact of peer support is not universally positive. Some studies, have found that peer support does not always facilitate improved mental well-being and can, in certain contexts, even be detrimental to positive affect (John et al., 2018). This complexity underscores the need to understand the dynamics of peer interactions and their psychological implications more deeply.

The relevance of peer support in the context of Social Identity Theory is particularly noteworthy. Social Identity Theory, a psychological framework, emphasizes the importance of group membership and social identities in shaping an individual’s behavior and attitudes. In the educational setting, a student’s identification with a peer group can significantly influence their academic engagement and personal development. A peer group not only provides emotional support for adolescents but also offers a social status necessary for identity development, a process especially crucial during the adolescent years (Ragelienė, 2016).

This theory’s application becomes even more critical in the context of English Language Learners (ELLs), particularly in countries like China. For ELLs, peer support can serve as a vital source of emotional and academic assistance, helping them navigate the challenges of learning a new language and adapting to a different cultural environment (Bíró et al., 2016). The sense of belonging and shared identity that peer groups provide can be instrumental in mitigating the feelings of isolation and alienation that ELLs might experience. It aids in their social integration and contributes positively to their language acquisition process (Basson and Rothmann, 2018).

In summary, Students’ Peer Support in educational settings, while complex and multifaceted, is a crucial element in fostering emotional health and engagement. Its significance is further amplified when considered through the lens of Social Identity Theory, especially for English Language Learners. This support system can be a powerful tool in enhancing the educational experience, promoting well-being, and supporting the overall development of students in diverse educational landscapes.

### 2.5 Mediating role of teachers’ harmony and peer’s support

The learning environment often functions as a complex “black box,” obscuring the processes that link predictive factors to educational outcomes. This necessitates a thorough analysis of the underlying mechanisms. There is empirical evidence indicating that Teachers’ Autonomy-Supportive Behavior significantly impacts students’ desired outcomes. However, the specific pathways through which Teachers’ Autonomy-Supportive Behavior exerts its influence are not fully understood.

Teachers’ Autonomy-Supportive Behavior may foster peer support, as this educational approach encourages individual initiative and a constructive motivation for learning and sharing positive experiences (Hofkens and Pianta, 2022). Simultaneously, Teachers’ Autonomy-Supportive Behavior can contribute to a more organized and structured classroom environment (Pastore and Luder, 2021). This, in turn, potentially reduces exhaustion and burnout among educators and promotes psychological well-being, as evidenced in constructs such as Teachers’ harmony (Han, 2021). In summary, by nurturing more autonomous and learning-oriented behaviors in students and by effectively structuring and organizing classroom activities, Teachers’ Autonomy-Supportive Behavior may positively influence student outcomes through the dual mediators of Teachers’ harmony and Peer support.

Based on the literature reviewed, we propose the following hypothesis:

*Hypothesis H1*. Teachers’ autonomy-supportive behavior, Students’ Peer support, and Teachers’ harmony significantly predict Students’ Emotional Health.

*Hypothesis H2*. Teachers’ autonomy-supportive behavior, Students’ Peer support, and Teachers’ harmony significantly predict Students’ Engagement.

*Hypothesis H3*. Teachers’ harmony and Students’ Peer support mediate the relationship between Teachers’ autonomy-supportive behavior and Students’ outcomes: both Emotional Health and Academic Engagement.

**Figure 1:**
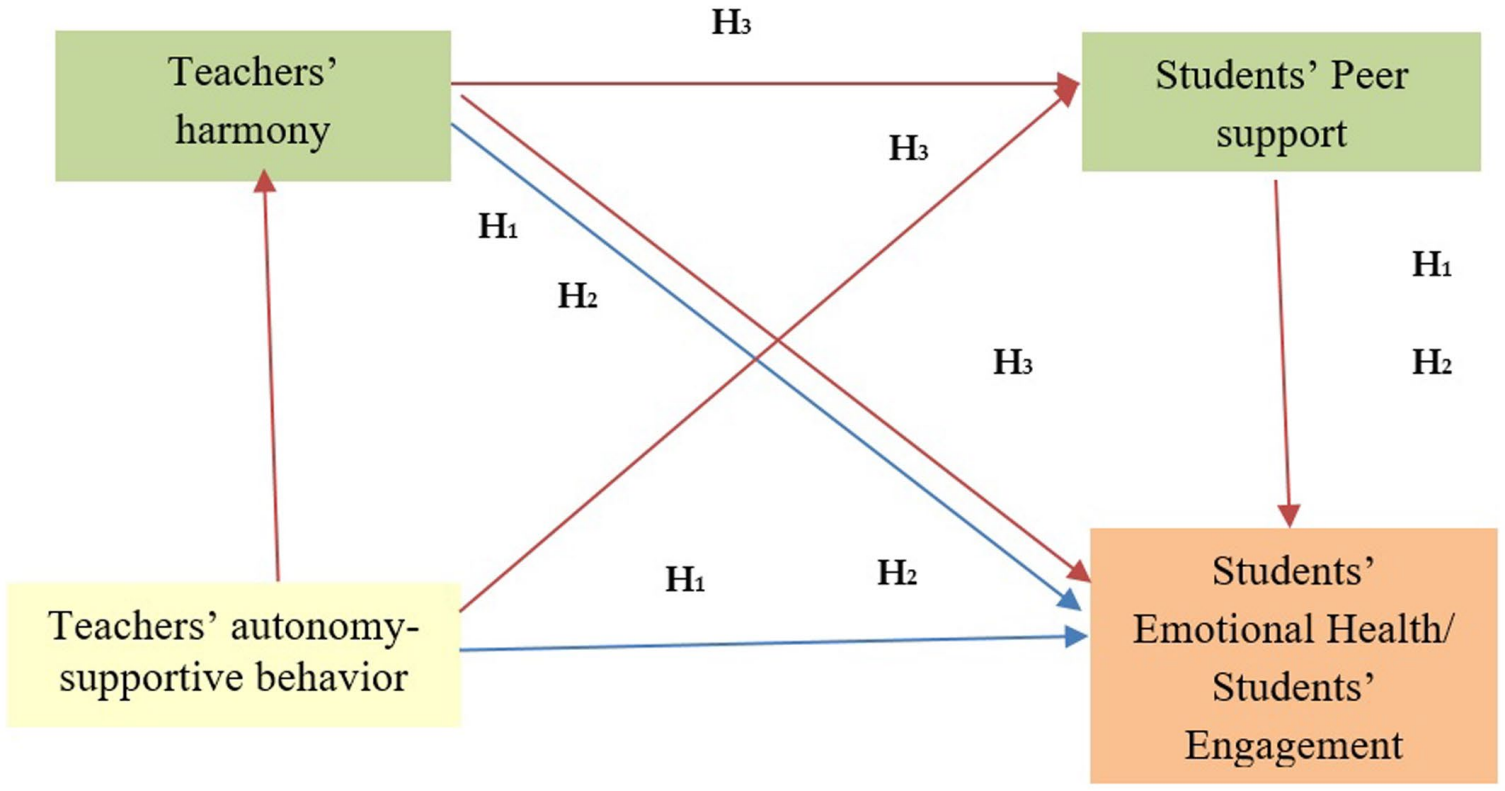
Mediating model of teachers’ harmony and students’ peer support, in the relationships between teachers’ autonomy-supportive behavior and students’ emotional Health/students’ engagement. β: Standardized coefficients and 95% Confidence Interval; *p < 0.05; **p < 0.01.

The conceptual model for the study is presented in Figure 1.

## 3 Methods

### 3.1 Sample and procedure

The current research involved participants who were Chinese English Language Teachers (*N* = 68) (Mean age = 40.68; S.D. = 10.17; 36.8% Males) and their corresponding English Language Students (*N* = 389), (Mean age = 21.86; S.D. = 2.53; 41.6% Males) drawn from the teachers’ classes. Regarding teaching seniority, 98.5% of the participants have been teaching more than five years, while the majority of students 62.7% were in their second year of University period. Related to the educational background, 52.9% of teachers had a Master’s Degree, and 26.5% had a Ph.D. Recruitment for both groups was facilitated through collaboration with students in a doctoral program specializing in English Language Teaching at various Chinese universities. To reach potential participants, emails were sent to institutions offering these programs, including but not limited to Xi’an Jiaotong-Liverpool University and the University of Nottingham Ningbo China. The invitation emails outlined the study’s purpose and specified the requirement for each participating teacher to involve a minimum of five of their students.

Those who consented to participate were subsequently sent informed consent forms and a link to the web-based survey. This study employed a convenience sampling method, with participation criteria that included being over 18 years old, actively teaching or studying English Language Teaching either full-time or part-time, having the ability to comprehend the survey, and having internet access. The data collection phase occurred throughout the year 2023 (April–May). To maintain confidentiality, the research team did not access any personal information of the participants. Ethical standards for the study conformed to the latest version of the World Medical Association’s Declaration of Helsinki, as revised in Fortaleza. Before being allowed to take part, each participant had to provide their informed consent. This consent was obtained via an online form hosted on the Qualtrics platform. The form clearly explained the aims of the research, emphasized that participation was entirely optional, and outlined the steps taken to protect the privacy of participants and the confidentiality of their data. The only information gathered was the commencement and completion dates of the survey, along with the percentage of the survey completed by each respondent. The ethical aspects of the study received approval from the Bio-Ethical Committee at Jinan University.

### 3.2 Measures

Survey participants were invited to express their level of agreement with items from various scales on a five-point scale, with 1 indicating strong disagreement and 5 indicating strong agreement. Higher scores were indicative of stronger agreement, without any reverse-scored items. Students answered the Teachers’ autonomy-supportive behavior, Students’ Peer support, Students’ Emotional Health, and Students’ Engagement, while Teachers only provided responses to the Teachers’ harmony. The Teachers’ punctuation on Teachers’ Harmony were assigned to the Students participants to conduct the analyses.

### 3.3 Teachers’ autonomy-supportive behavior

To evaluate students’ views on their teachers’ autonomy-supportive actions, the Learning Climate Questionnaire, developed by Black & Deci, was employed (Black and Deci, 2000). This tool consists of six questions. The average of the eight item scores was calculated to obtain the Teachers’ Autonomy-Supportive Behavior score. This suggests a greater perception of teacher autonomy support. For instance, one item from the questionnaire is, “My teacher gives me various options and choices.” In this study, the LCQ showed good reliability (Cronbach’s Alpha = 0.72).

### 3.4 Students’ peer support

The cooperation aspect of peer support among students was measured using the cooperation subscale, with eight items, from the “What Is Happening in this Class?” (WIHIC) questionnaire, originally developed by Fraser & Fisher, and later used by Fraser and Fisher (1983) and Skordi and Fraser (2019). This included items like “I share my resources with others during assignments” and “We demonstrate teamwork in group activities in this class.” (Cronbach’s Alpha = 0.80).

### 3.5 Teachers’ harmony

Teachers’ psychological harmony was measured using the Psychological Harmony Scale, a Chinese-developed instrument by Ren et al. in 2009. The scale originally comprised 44 items across four dimensions of harmony (Ren et al., 2009). In this study, four items were included. These items were the most representative item from each dimension, covering aspects such as personal contentment (“Overall, I am satisfied with my life”), family atmosphere (“My family is very harmonious”), interpersonal relations (“The people around me help each other”), and social connectivity (“I have a good number of social connections”). The higher score indicates a better psychological harmony. The reliability of the original scale was high, with a Cronbach’s alpha of 0.85 (Chu and Gong, 2023), while in the present study, the reduced version reached a Cronbach’s Alpha = 0.70.

### 3.6 Students’ emotional health

The Positive and Negative Affect Scale (PANAS), developed by Watson et al., was utilized to explore the relationship between the predictors in the MIMIC model and students’ emotional health (Watson et al., 1988). This scale assesses recent emotional experiences over a two-week period using a five-point response scale. The current study employed an abbreviated version of PANAS with four items exhibiting high factor loadings as identified by Crawford and Henry (2004), focusing on positive affect (such as feeling alert, inspired, determined, and attentive).

### 3.7 Students’ engagement

To measure student engagement, the short form of the Utrecht Work Engagement Scale (UWES–3) by Schaufeli et al. (2017) was used, adapted to the context of academic activities as previously done in studies like that of Dominguez-Lara et al. (2021). This scale encompasses three items, each representing a dimension of engagement: vigor, dedication, and absorption, with examples such as feeling energetic about studies, being inspired by studies, and being engrossed in studying. The reliability of these scale was satisfactory, with Cronbach’s alpha values of 0.82.

### 3.8 Data analyses

Given that students were assigned to the classrooms, and the Teacher’s harmony punctuations have been assigned to each class, intra-class correlation coefficients (ICC) was calculated to obtain the extent to which individual differences originate from class differences, as several studies done (Zheng et al., 2023). As a rule of thumb for interpretation (Cohen, 1977), if the ICC is lower than 0.059, it indicates that there are not class differences to justify an Hierarchical Linear Model utilization. In our case, none of the values of ICC were larger than the cut of point. The analyses have been carried out with the Seolmatrix, a R module for conducting ICC analyses (Gamer et al., 2019). The following analysis utilized JASP software, version 0.18.1, to conduct statistical assessments. Two distinct models were evaluated: the first employing UWES as the key indicator, while the second used Emotional Health as the primary indicator. Common predictors in both models included Teachers’ autonomy-supportive behavior, Teachers’ sense of harmony, and Peer support. The fit of the model was determined using several metrics as recommended by various scholars. These included the Chi-square value and its corresponding value of p, the Adjusted Goodness of Fit Index (AGFI), Comparative Fit Index (CFI), Normed Fit Index (NFI), and Incremental Fit Index (IFI). It was noted that for these indices, values exceeding 90 suggest a robust fit. Conversely, the Root Mean Square Error of Approximation (RMSEA) and Standardized Root Mean Square Residual (SRMSR) were deemed acceptable at values below 0.08, with figures around 0.06 indicating an exceptionally strong fit between the model and the observed data.

Additionally, a MIMIC Model, akin to a Structural Equation Model, was applied to evaluate the initial hypotheses. The MIMIC Model was preferred for two main reasons. Firstly, it provides a measurement of the intention to travel (used here as the dependent variable) that is free from errors. Secondly, it allows for the examination of unique relationships within the dimensions of job crafting indicator items. The maximum likelihood estimation method was chosen for this analysis, with a focus solely on the standardized coefficients within the model. The assumption of normality was verified using Kolmogorov’s test, with a significance level set above 0.05.

To test the third hypothesis regarding the mediating role of both Peers’ support and Teachers’ harmony the Model 6 of PROCESS macros for SPSS has been used (Hayes, 2022). The Model 6 estimates the indirect effect of X (Teachers’ autonomy-supportive behavior) on Y (Students’ Emotional Health or Students’ Engagement) through M1 (Students’ Peer support), and M2 (Teachers’ harmony). This procedure was based on 5,000 bootstrap re-samples and provided estimates of the indirect effect and associated confidence intervals. When zero is not included in the 95% bias-corrected confidence interval, it may be concluded that the parameter is significantly different from zero at *p* < 0.05.

## 4 Results

Table 1 shows the Pearson’s correlations between the indicators (Students’ Engagement, and Emotional health) with the predictors of the MIMIC model. Thus, all correlations were significant (*p* < 0.001) excepts Teacher’s harmony–Students’ Emotional health (*r* = 0.101, *p* < 0.05) and Teachers’ autonomy-supportive behavior–Students’ Emotional health (*r* = 0.0055, *p* > 0.05).

**Table 1:** Pearson correlation between students’ emotional health and students’ engagement and predictors of the MIMIC.

Variables	*M*	*S.D.*	1	2	3	4
1. Teachers’ autonomy-supportive behavior	3.740	0.679	—			
2. Students’ Peer support	3.752	0.562	0.028	—		
3. Teachers’ harmony	4.040	0.570	0.296***	0.384***	—	
4. Students’ emotional health	3.934	0.807	0.055	0.195***	0.101*	—
5. Students’ engagement	4.052	0.694	0.314***	0.192***	0.296***	0.095

*p < 0.05; ***p < 0.001. *N* = 389.

Multiple indicator multiple cause analyses.

First, the model to predict Students’ Emotional Health showed an optimal fit with the data: *X*^2^ = 29.32, *p* < 0.01, CFI = 0.945, NFI = 0.910, TLI = 0.910, RMSEA = 0.06 and SRMR = 0.03. The amount of variance explained was *R*^2^ = 0.05. Students’ Emotional Health was predicted only by Students’ Peer support (*B* = 0.354, *p* < 0.01). The rest of variables included in the model were not statistically related with the job crafting interest (*p* > 0.05).

The second model predicting Students’ Engagement showed an optimal fit with the data: *X*^2^ = 19.14, *p* < 0.01, CFI = 945, NFI = 0.900, TLI = 0.900, RMSEA = 0.06 and SRMR = 0.03. The amount of variance explained was *R*^2^ = 0.277. Students’ Engagement was predicted the three variables in the model: Teachers’ autonomy-supportive behavior (*B* = 0.627, *p* < 0.01), Students’ Peer support (*B* = 0.258, *p* < 0.01), and Teachers’ Harmony (*B* = 0.487, *p* < 0.01).

### 4.1 Mediation analyses

The direct effect of Teachers’ Autonomy-Supportive Behavior on Students’ Emotional Health was positive but not significant (c’ = 0.049, *p* = 0.34). The direct model was significant (*F* (3,385) = 28.50, *p* < 0.001, *R*^2^ = 0.18). The total effect was positive and statistically significant (c = 0.17, *p* < 0.01), and the overall model was significant (*F* (1,387) = 18.17, *p* < 0.01, *R*^2^ = 0.05), providing only partial support for Hypothesis 3.

The direct effect of Teachers’ Autonomy-Supportive Behavior on Students’ Engagement was positive but not significant (c’ = 0.106, *p* = 0.08). The direct model was significant (*F* (3,385) = 9.48, *p* < 0.001, *R*^2^ = 0.07). The total effect was positive and statistically significant (c = 0.19, *p* < 0.01), and the overall model was significant (*F* (1,387) = 12.25, *p* < 0.01, *R*^2^ = 0.03), again providing only partial support for Hypothesis 3.

To test Hypothesis 3, three indirect effects were considered in predicting Students’ Emotional Health. Firstly, one of them (Indirect effect No. 2) was not significant, as shown in Table 2. Secondly, two of them (Indirect effect No. 1, and 3) were significant. The indirect effects through Teachers’ Harmony (No. 1), and the combination of Teachers’ Harmony and Students’ Peer Support (No. 3), displayed statistical significance, thereby supporting Hypothesis 3.

**Table 2:** Completely standardized indirect effect(s) of X on Y.

	Effect	BootSE	BootLLCI	BootULCI
*Outcome variable (Y): students’ emotional health*				
TOTAL	0.1625	0.0369	0.0946	0.2382
Indirect 1	0.1220	0.0294	0.0672	0.1854
Indirect 2	−0.0051	0.0149	−0.0363	0.0245
Indirect 3	0.0456	0.0135	0.0207	0.0745
*Outcome variable (Y): students’ engagement*				
TOTAL	0.0783	0.0242	0.0320	0.1269
Indirect 1	0.0530	0.0231	0.0080	0.0990
Indirect 2	−0.0032	0.0096	−0.0220	0.0176
Indirect 3	0.0284	0.0116	0.0075	0.0535
*Indirect effect key:*				
Indirect 1 Teachers’ autonomy-supportive behavior → teachers’ harmony → students’ emotional health or students’ engagement				
Indirect 2 Teachers’ autonomy-supportive behavior → students’ peer support → students’ emotional health or students’ engagement				
Indirect 3 Teachers’ autonomy-supportive behavior → teachers’ harmony → students’ peer support → students’ emotional health or students’ engagement				

In the context of predicting Students’ Engagement for testing Hypothesis 3, three indirect effects were evaluated. Firstly, one was not significant (Indirect effect No. 2), as indicated in Table 2. Secondly, the other two (Indirect effect No. 1, and 3) were significant. The indirect effects through Teachers’ Harmony (No. 1), and the combination of Teachers’ Harmony and Students’ Peer Support (No. 3), showed statistical significance, supporting Hypothesis 3.

**Figure 2:**
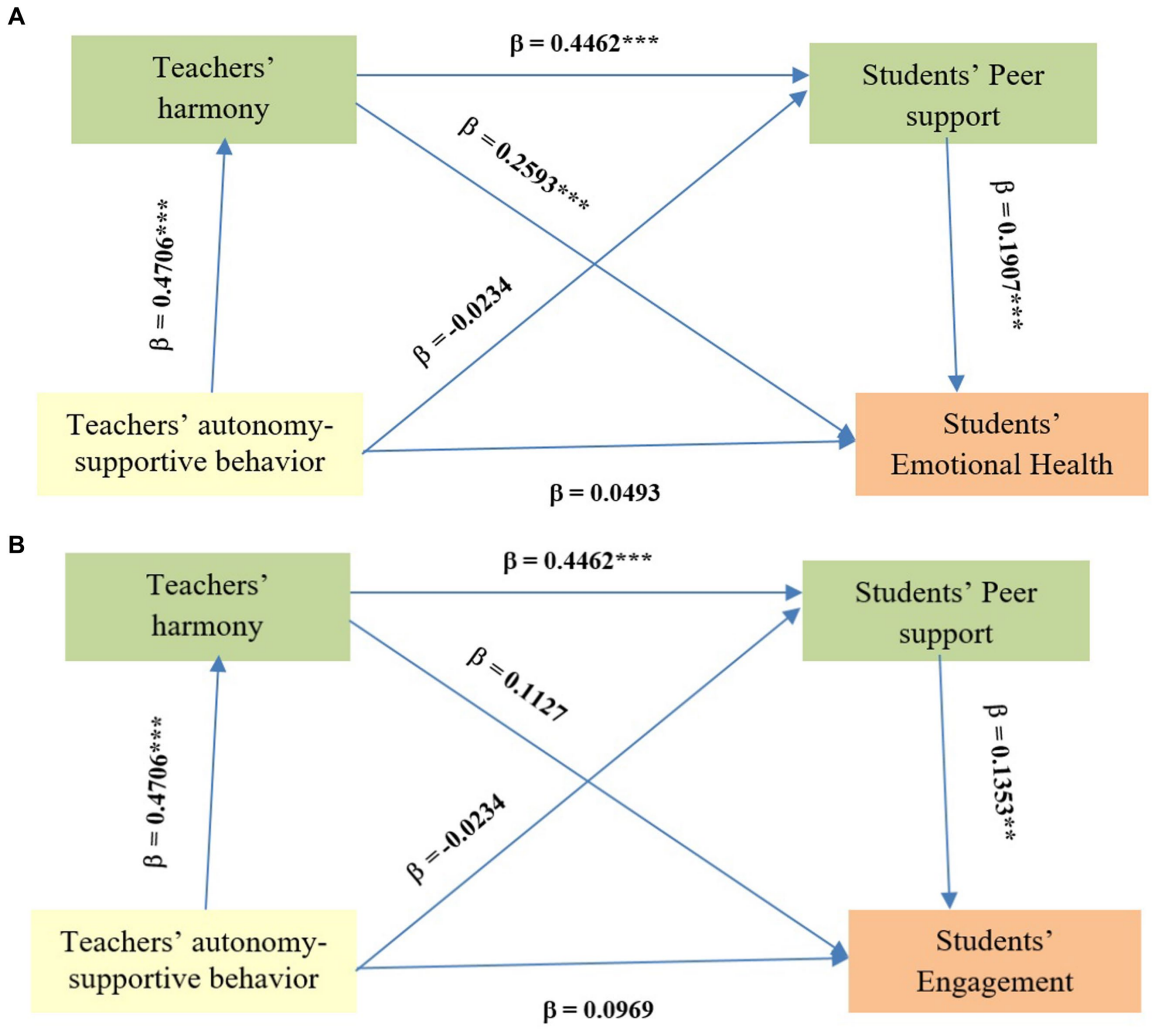
Mediating model of students’ peer support, and teachers’ harmony in the relationships between teachers’ autonomy-supportive behavior and students’ **(A)** emotional health and **(B)** engagement. β: Standardized coefficients and 95% Confidence Interval; *p < 0.05; **p < 0.01.

Standardized effects are shown in Figures 2A,B.

## 5 Discussion

The study’s first model tested by the MIMIC procedure, focusing on predicting students’ emotional health, demonstrated an optimal fit. Notably, only peer support emerged as a significant predictor (*B* = 0.354, *p* < 0.01). This finding aligns with previous research underscoring the critical role of peer support in influencing emotional well-being in educational settings. However, it partially diverges from studies like those by Vargas-Madriz and Konishi (2021), which found a more robust influence of teacher-related factors on student emotional health. This discrepancy might be attributed to cultural variations or differences in educational systems, suggesting that the impact of teachers and peers on students’ emotional health can vary significantly across contexts.

The second model, predicting students’ engagement, revealed that all three variables—teachers’ autonomy-supportive behavior, peer support, and teachers’ harmony—were significant predictors. The strong influence of teachers’ autonomy-supportive behavior (*B* = 0.627, *p* < 0.01) is consistent with the self-determination theory (Ryan and Deci, 2000), which posits that autonomy support is crucial for fostering engagement and intrinsic motivation in learners. This result is also in line with previous findings (Yang et al., 2022), emphasizing the importance of teacher behavior in enhancing student engagement.

Interestingly, the study also found a substantial effect of teachers’ harmony on student engagement (*B* = 0.487, *p* < 0.01), a variable less explored in existing literature. This finding suggests a unique contribution of the relational and emotional climate set by teachers to the engagement levels of students, echoing the relational-cultural theory (Ren et al., 2009) that emphasizes the significance of relationships and connectedness in educational settings.

In contrast, the lower predictive power of peer support for engagement (*B* = 0.258, *p* < 0.01), compared to teachers’ autonomy-supportive behavior and harmony, suggests a more nuanced role of peer interactions in fostering engagement. This finding partially aligns with studies by Sadoughi and Hejazi (2023), which indicated that while peer support is essential, it might not be as influential as the teacher-student dynamic in educational outcomes like engagement.

The third hypothesis of the present study, tested by the Model 6 of PROCESS, obtained limited support. Firstly, the direct effect of Teachers’ autonomy-supportive behavior both on Students’ Emotional Health and on Students’ Engagement was positive but not significant. The indirect effects that reached statistical significance were those through Teachers’ Harmony (No. 1), and the combination of Teachers’ Harmony and Students’ Peer Support (No. 3), both on Students’ Emotional Health and Engagement. The present results confirmed the relevance of psychological harmony as a powerful variable that accounts for wellbeing in whole life (Guo-Xin, 2013), as well as in the workplace activities, such as authors stated (Di Fabio and Tsuda, 2018). At the same time, potential influence of Teachers’ psychological Harmony, not only on their own wellbeing, but also on students’ outcomes, agreed with previous findings and suggestions (Tharp et al., 2018; Uwayid and Jabbar, 2023). In this vein, some professional development initiatives are proposed for increasing Teachers’ harmony that could, in turn, have an impact on learners’ (Hadar and Brody, 2010; Baumfield et al., 2023; Wang and Zou, 2023).

In summary, this study contributes to the understanding of factors influencing emotional health and engagement in English language learners. It highlights the paramount importance of peer support for emotional health and delineates a more complex interplay of teacher behavior and peer interactions in fostering student engagement. These insights underscore the need for a holistic approach in educational interventions focusing on both teacher and peer dynamics to enhance emotional health and engagement among learners.

### 5.1 Limitations of the present research and suggestions for future studies

Several key points need to be addressed. These limitations stem primarily from the methodology and sampling approach, as well as the measures and analytical techniques used.

Firstly, the use of convenience sampling limits the generalizability of the findings. Since the participants were recruited from specific universities through collaboration with doctoral students, the sample may not be representative of the broader population of English language teachers and students in China. This selection bias could influence the study’s results, as participants from these specific educational settings might have unique characteristics or experiences that are not indicative of the larger population.

Secondly, the reliance on self-report measures could lead to response bias. Participants’ responses on surveys are subject to their perceptions and interpretations. This subjective nature can impact the accuracy and reliability of the data collected, and combined with the cross-sectional design, could be considered the main limitation of the present research.

Furthermore, the study’s analytical approach, while robust, has its limitations. The use of the MIMIC model, though advantageous for error-free measurement and unique relationship examination, might not capture the full complexity of the interactions among the variables studied. The assumption of normality, verified using Kolmogorov’s test, may not hold true in all cases, potentially affecting the findings’ validity.

The limited variance explained in some of the models, particularly the one predicting students’ emotional health, suggests that there are other factors not included in the study that significantly influence these outcomes. This indicates a need for more comprehensive models that incorporate additional variables potentially affecting emotional health and engagement.

Finally, the study’s cross-sectional design limits its ability to establish causality. While the relationships identified are significant, they do not necessarily imply cause and effect. Longitudinal studies would be required to better understand the causal relationships between teachers’ autonomy-supportive behavior, teachers’ harmony, peer support, and the emotional health and engagement of English language learners.

These limitations should be considered when interpreting the study’s findings and in the design of future research in this area.

### 5.2 Practical implications and suggestion for educational interventions

In addressing the practical implications and suggestions for educational intervention derived from our study, it becomes clear that the role of teachers and peers is pivotal in enhancing both the emotional health and engagement of English language learners in China. Our research has illuminated the significant impact of teacher autonomy-supportive behavior and peer support in the educational setting, offering valuable insights for developing more effective teaching strategies and learning environments.

A key implication of our findings is the necessity for educational institutions to prioritize the enhancement of teacher autonomy-supportive behavior. This could involve the implementation of professional development programs specifically designed to equip teachers with skills in autonomy-supportive teaching practices. Such training would ideally focus on strategies that enable teachers to provide more choices to students, acknowledge their feelings, and encourage self-initiative and independent thinking (Tharp et al., 2018). In this vein, teachers’ professional development through learning community initiatives could help them in reducing professional burnout (Chu and Gong, 2023) by increasing psychological harmony (Hadar and Brody, 2010). Additionally, the creation of forums or workshops where teachers can exchange best practices and learn from one another could further foster an environment of autonomy and support in the classroom (Zhang and Zhou, 2008). In a related vein, the inclusion of students’ perspectives on these processes could enhance the relevance and effectiveness of the strategies implemented.

Simultaneously, the importance of peer support in both emotional health and student engagement cannot be overstated. Schools and educational programs should actively work toward creating a classroom culture that encourages collaboration and mutual support among students. This could be achieved through activities and teaching methods that promote teamwork and peer interaction, thereby enhancing the learning experience and building a strong sense of community within the classroom.

Furthermore, the concept of teachers’ harmony, though not the primary focus of our study, has emerged as a potentially influential factor in student engagement. This suggests that teacher training programs should not only focus on teaching methodologies but also include components aimed at improving personal well-being and interpersonal harmony (Kim and Plester, 2019). Incorporating training in areas such as conflict management (Donnelly, 2004), emotional intelligence (Al-Rabadi, 2012), and effective communication could prove beneficial in creating a more harmonious and productive learning environment, helping students to cope with aggressive behavior and anger (Tao et al., 2020).

Moreover, the design of the curriculum and the choice of instructional strategies are crucial in actualizing these findings. Integrating project-based learning and problem-solving tasks that necessitate student collaboration can not only enhance academic outcomes but also foster peer support, autonomy, and engagement (Wang and Zou, 2023). This approach to curriculum design, coupled with the effective use of educational technology, could significantly enrich the learning experience and promote a supportive and engaging educational environment. In designing interventions, cultural context and educational settings diversity should be taken into account for adapting the recommendations to different audiences. Following this idea, intervention effectiveness needs to be assessed, as well as potential limitations in implementation of programs recognized, in order to maximize the applicability of the designs and to cope with scarcity of resources that precludes success.

In conclusion, our study underscores the need for a holistic approach in educational interventions, focusing on both teacher development and the cultivation of a supportive peer environment. By addressing these key areas, we can move toward creating more effective and emotionally supportive educational settings for English language learners.

## 6 Conclusion

In concluding, the present research has made significant contributions to the understanding of factors that influence the emotional health and engagement of English language learners. The study’s results indicate that students’ peer support plays a critical role in predicting their emotional health. This finding underscores the importance of a supportive peer environment in educational settings, highlighting how peer interactions can significantly impact the emotional well-being of students.

Furthermore, the study revealed that students’ engagement is influenced not only by peer support but also by teachers’ autonomy-supportive behavior and the concept of teachers’ harmony. The strong predictive power of teachers’ autonomy-supportive behavior on student engagement aligns with the principles of self-determination theory. It emphasizes the importance of teacher behavior in creating an educational environment that fosters student motivation and engagement. The influence of teachers’ harmony, while less explored in the literature, emerged as a significant factor, suggesting that the emotional and relational climate established by teachers is crucial for student engagement.

The methodological approach of the study, employing a MIMIC model and utilizing various reliable measures, provided robust insights into these relationships. However, the limitations inherent in the study’s design, particularly the use of convenience sampling and self-report measures, highlight the need for cautious interpretation of the findings and suggest areas for further research.

The practical implications of these findings are manifold. They suggest that interventions aimed at improving student outcomes in English language learning should not only focus on pedagogical strategies but also on fostering a supportive peer environment and enhancing teacher-student relationships. Professional development for teachers focusing on autonomy-supportive teaching practices and personal well-being, along with initiatives to encourage positive peer interactions among students, are essential for creating an effective and emotionally healthy learning environment.

In conclusion, this study contributes valuable insights into the dynamics of teacher and peer influences on the emotional health and engagement of English language learners. It underscores the need for a holistic approach in educational settings that considers both the role of the teacher and the peer group in fostering an environment conducive to learning and emotional well-being. As the field of English language education continues to evolve, these findings provide a foundation for future research and practice aimed at enhancing the educational experiences of learners. To sum up, the present findings offer relevant insights into the subtle process that links Teachers’ autonomy-supportive behavior and students’ outcomes, through Teachers’ harmony, providing and empirically informed approach for improving other educational experiences with ELLs.

## Data availability statement

The raw data supporting the conclusions of this article will be made available by the authors, without undue reservation.

## Ethics statement

The studies involving humans were approved by Bio-Ethical Committee at Jinan University. The studies were conducted in accordance with the local legislation and institutional requirements. The participants provided their written informed consent to participate in this study.

## Author contributions

LY: Writing – original draft, Writing – review & editing.
